# From Function to Metabolome: Metabolomic Analysis Reveals the Effect of Probiotic Fermentation on the Chemical Compositions and Biological Activities of *Perilla frutescens* Leaves

**DOI:** 10.3389/fnut.2022.933193

**Published:** 2022-07-11

**Authors:** Zhenxing Wang, Ximeng Jin, Xuechun Zhang, Xing Xie, Zongcai Tu, Xiahong He

**Affiliations:** ^1^Key Laboratory for Forest Resources Conservation and Utilization in the Southwest Mountains of China, Ministry of Education, Southwest Forestry University, Kunming, China; ^2^College of Life Sciences, Southwest Forestry University, Kunming, China; ^3^National R&D Center for Freshwater Fish Processing, College of Health, Jiangxi Normal University, Nanchang, China; ^4^College of Horticulture and Landscape, Southwest Forestry University, Kunming, China

**Keywords:** *Perilla frutescens* leaves, fermentation, phytochemical composition, biological activity, metabolomics

## Abstract

This study aimed to investigate the impact of probiotic fermentation on the active components and functions of *Perilla frutescens* leaves (PFL). PFL was fermented for 7 days using six probiotics (*Lacticaseibacillus Paracasei* SWFU D16, *Lactobacillus Plantarum* ATCC 8014, *Lactobacillus Rhamnosus* ATCC 53013, *Streptococcus Thermophilus* CICC 6038, *Lactobacillus Casei* ATCC 334, and *Lactobacillus Bulgaricus* CICC 6045). The total phenol and flavonoid contents, antioxidant abilities, as well as α-glucosidase and acetylcholinesterase inhibition abilities of PFL during the fermentation process were evaluated, and its bioactive compounds were further quantified by high-performance liquid chromatography (HPLC). Finally, non-targeted ultra-HPLC–tandem mass spectroscopy was used to identify the metabolites affected by fermentation and explore the possible mechanisms of the action of fermentation. The results showed that most of the active component contents and functional activities of PFL exhibited that it first increased and then decreased, and different probiotics had clearly distinguishable effects from each other, of which fermentation with ATCC 53013 for 1 day showed the highest enhancement effect. The same trend was also confirmed by the result of the changes in the contents of 12 phenolic acids and flavonoids by HPLC analysis. Further metabolomic analysis revealed significant metabolite changes under the best fermentation condition, which involved primarily the generation of fatty acids and their conjugates, flavonoids. A total of 574 and 387 metabolites were identified in positive ion and negative ion modes, respectively. Results of Spearman’s analysis indicated that some primary metabolites and secondary metabolites such as flavonoids, phenols, and fatty acids might play an important role in the functional activity of PFL. Differential metabolites were subjected to the KEGG database and 97 metabolites pathways were obtained, of which biosyntheses of unsaturated fatty acids, flavonoid, and isoflavonoid were the most enriched pathways. The above results revealed the potential reason for the differences in metabolic and functional levels of PFL after fermentation. This study could provide a scientific basis for the further study of PFL, as well as novel insights into the action mechanism of probiotic fermentation on the chemical composition and biological activity of food/drug.

## Introduction

Fermentation is one of the classic methods of food processing. Under the effects of microorganisms, food components are transformed and degraded, resulting in a variety of secondary metabolites, which in turn have positive impacts on food quality, such as flavor, taste, nutritional value, functional properties, and shelf-life (1).

Due to the complexity of the food system, these quality characteristic changes in food and its underlying mechanisms during fermentation have been an important research focus. In recent years, metabolomics, including liquid chromatography-tandem mass spectrometry (LC-MS/MS), gas chromatography-tandem mass spectrometry (GC-MS/MS), and nuclear magnetic resonance (NMR), has been widely used in investigating the chemical composition and metabolite contents of food products (2). They provide fast and sensitive methods for the identification and quantification of the small molecules produced in food processing, which could be used to well explain the changes in product, flavor, and nutrition during the fermentation process (3, 4). However, in-depth studies on metabolite changes in fermented food and their association with functional activities are still lacking.

*Perilla frutescens* (L.) Britt. is a traditional food and medicinal plant and is widely cultivated and distributed in many East Asian countries (5). As its primary edible relevant part, the *perilla frutescens* leaf (PFL) is widely used for fresh vegetables, condiments, and kimchi. In addition, its medicinal properties have been proven in traditional medicine for centuries, such as diuresis, antitussive, detoxification, diaphoretic, etc. (6, 7). Modern pharmacological research has revealed that PFL exerts diverse effects on chronic diseases related to oxidative stress, such as diabetes, cancer, inflammatory, hypertension, Alzheimer’s disease, etc. (8–11). Literature and our previous work (12, 13) indicated that PFL possessed excellent biological activities including antioxidant, anticancer, antibacterial, and anti-inflammatory activity, which was associated with their abundant active ingredients (e.g., phenolics and flavonoids) (14, 15). PFL has been considered to be an excellent raw material for fermented foods. Research shows that the nutritional value and biomedical benefits of *Perilla frutescens* seeds are enhanced by fermentation, although there are only a few studies on PFL fermentation, and the dynamic changes of its active components and functional activities in the fermentation process are not clear (16).

In this study, we employed six different probiotics to ferment PFL. The differences and variations in the chemical composition and functional activities of the fermented *perilla frutescens* leaf (FPFL) were evaluated by spectrophotometry and high-performance liquid chromatography (HPLC) methods, thus selecting the best performing strain and elucidating the dynamic changes of the ingredients and activities during the fermentation process. Then we characterized the metabolic transition before and after fermentation using mass spectrometry-based metabolomics. By combining mass-spectrometry metabolomics with the above *in vitro* study, the main compounds related to the functional activities of PFL were obtained. Finally, KEGG pathway enrichment analysis of the differential metabolites was performed to investigate the possible action mechanism of probiotic fermentation on PFL. We expect that our results could provide a novel insight into the biotransformation of the active components in natural products/foods and the scientific basis for the further development and utilization of *Perilla frutescens*.

## Materials and Methods

### Chemicals and Reagents

*Lacticaseibacillus Paracasei* SWFU D16 was isolated from Yunnan goat milk cake, a Chinese traditional fermented food in our Laboratory. *Lactobacillus Plantarum* ATCC 8014, *Lactobacillus Bulgaricus* CICC 6045, *Lactobacillus Casei* ATCC 334, and *Streptococcus Thermophilus* CICC 6038 were purchased from the Guangdong Microbial Culture Collection Center (GDMCC), and *Lactobacillus Rhamnosus* ATCC 53013 was purchased from the American Type Culture Collection (ATCC).

Folin–Ciocalteu reagent, gallic acid, rutin, 2,2-diphenyl-1-picrylhydrazyl radical (DPPH), 2,2’-azinobis (3-ethylbenzothiazoline-6-sulfonic acid) diammonium salt (ABTS), 2,4,6-tri (2-pyridyl)-1,3,5-triazine (TPTZ), iron chloride (FeCl_3_6H_2_O), p-Nitrophenol α-D-glycopyranoside (pNPG), acetylthiocholine iodide (ATCI), 5,5’-Dithiobis-(2-nitrobenzoic acid) (DTNB), and other chemicals were purchased from Aladdin (Shanghai, China). Chromatographic acetonitrile was purchased from Merck (Darmstadt, Germany). α-Glucosidase (G5003), and acetylcholinesterase (AChE, C3389) were purchased from Sigma-Aldrich (St. Louis, United States). Syringic acid, (+)-catechin, rosmarinic acid, chlorogenic acid, and other standards were purchased from Yuanye Bio-Technology (Shanghai, China).

### Material Preparation and Fermentation

Mature PFL were purchased from Meizhou city, Guangdong province, China. After drying at 50 °C (Thermostatic blast drying, DHG Series, Shanghai, China), samples were pulverized and sifted through a 40-mesh sieve, then mixed with distilled water in a 1:25 ratio (mass/volume) in a conical flask, and autoclaved at 121°C for 15 min. When cooled to room temperature, 10 mL of probiotics (1.5 × 10^7^ CFU) were added and incubated at 37°C for 7 days. For consistency, each probiotic was from the same culture bottle. In parallel, 2 g glucose and 2 g skimmed milk powder were added as the carbon and nitrogen source (CN) controls, respectively. During the fermentation, fermented *Perilla frutescens* leaves (FPFL) were sampled daily and frozen at -18°C pending determination.

### pH Values

The pH was measured by directly placing a pH electrode of the pH meter (Hanna Instrument, Ann Arbor, Michigan, United States) into samples at room temperature.

### Total Phenolic and Flavonoid Content

Total phenolic content (TPC) was quantified by the Folin–Ciocalteau method with some modifications (17). Briefly, 40 μL of properly diluted sample was mixed with 20 μL of Folin–Ciocalteau reagent (0.5 M) in a 96-well microplate and incubated for 5 min. Next, 160 μL of Na_2_CO_3_ (7.5%, w/v) was added to the mixtures. The reaction was then kept in the dark for 30 min at room temperature, after which the absorbance was measured at 765 nm. As for the standard, gallic acid was used, and the data were provided as milligram gallic acid equivalent (mg GAE/g sample). For easier comparison, the final TPC results were expressed as the relative content (%) compared with the equivalent value of FPFL at the 0 days, and the starting TPC was 100%.

Total flavonoid content (TFC) was measured by the aluminum nitrate colorimetric method (18). A total of 20 μL of NaNO_2_ (3%, w/v) was mixed with 40 μl of a properly diluted sample. After 6 min of reaction, 20 μL of Al (NO_3_)_3_ (6%, w/v) was added after 6 min of incubation, and the mixture was incubated for another 6 min. Subsequently, 140 μL of NaOH (4%, w/v) and 60 μL of 70% methanol were added. The mixture solution stood for 15 min and the absorbance was measured at 510 nm. Rutin was used as a standard, and the data were calculated as milligram rutin equivalent per gram of sample (g RE/g sample). Similarly, the TFC was also expressed as a relative content (%).

### Antioxidant Assays

The abilities to scavenge DPPH and ABTS radicals were estimated by following the methods of Dong (19) and Wang (12) with slight modifications. Briefly, samples (100 μL) and 100 μL of DPPH (0.15 mM) were added to the 96-well microplate. The mixture was shaken thoroughly and then kept for 30 min in the dark at room temperature. Subsequently, the absorbance was measured at 517 nm. In the ABTS assay, 50 μL of samples were added to 200 μL of ABTS⋅^+^ freshly prepared working solution in a 96-well microplate and incubated for 6 min in the dark, and then the absorbance was measured at 734 nm. The sodium phosphate buffer (pH 6.9) was used instead of the DPPH or ABTS⋅^+^ solution as the control, and sodium phosphate buffer instead of the sample was used as the blank. The percentage of scavenging was calculated following Formula (1). The radical scavenging abilities of DPPH and ABTS were expressed as a relative percentage (%) compared with the scavenging rate of FPFL on the 0th day.


(1) $$Scavengingrate\left(\%\right)=\left(1-\frac{A_{s\,a\,m\,p\,l\,e}-A_{c\,o\,n\,t\,r\,o\,l}}{A_{b\,l\,a\,n\,k}}\right)\times 100$$

The ferric reducing antioxidant power (FRAP) was quantified by the reported method (20). The FRAP solution was prepared with 1 ml of TPTZ (7 mM), 1 mL of FeCl_3_ (20 mM), and 10 mL of acetate buffer (pH 3.6). Properly diluted samples (50 μL) were mixed with 200 μL of freshly prepared FRAP working solution in a 96-well microplate. After incubation in darkness for 10 min at 37°C, the absorbance was measured at 593 nm. The sodium phosphate buffer (pH 6.9) was used instead of the FRAP solution as the control, FeSO_4_ was used as the standard, and the data were calculated as milligram of FeSO_4_ equivalent per gram of sample (g FeSO_4_/g sample). FRAP was expressed as the relative percentage (%) compared with the equivalent value of FPFL at the 0 days.

### α-Glucosidase Inhibition Ability

The α-glucosidase inhibition ability was determined according to the previous method (21). Briefly, 50 μL of the samples were added to 50 μL of 0.1 U/mL α-glucosidase solutions and mixed in a 96-well plate. After incubation at 37°C for 10 min, a 50 μL of 5 mM pNPG solution was added and reacted for 15 min at 37°C. Finally, 100 μL of Na_2_CO_3_ (0.2 M) was added to terminate the reaction and the absorbance was determined at 405 nm. The sodium phosphate buffer (pH 6.9) was used instead of the α-glucosidase solution as control, and sodium phosphate buffer instead of the sample was used as the blank. The percentage of inhibition was calculated following Formula (1). The results were expressed as the relative percentage (%) compared with the inhibition rate of FPFL on the 0th day.

### Acetylcholinesterase Inhibition Ability

The acetylcholinesterase (AChE) inhibition ability was assessed using a colorimetric method (22). Initially, 50 μL of samples, 15 μL of ATCI (15 mM), and 75 μL of DTNB (3 μM) were mixed in a 96-well plate and incubated for 10 min at 30°C. Then 20 μL of 0.1 U/mL AChE and 50 μL of sodium phosphate buffer (pH 8.0) were added and shaken for 10 s. Followed by exposure to blocking light for 30 min at room temperature, the absorbance was measured at 410 nm by using a microplate reader. The sodium phosphate buffer (pH 6.9) was used instead of the AChE solution as the control, and sodium phosphate buffer instead of the sample was used as the blank. The percentage of inhibition was calculated following Formula (1). The results were expressed as the relative percentage (%) compared with the inhibition rate of FPFL on the 0th day.

### High-Performance Liquid Chromatography-DAD Analysis

HPLC-DAD analysis was performed by an HPLC 1260 (Agilent Technologies, CA, United States) equipped with a degasser, quaternary pump solvent delivery, thermo-stated column compartment, and a diode array detector. Samples were filtered through 0.22-μm nylon syringe filters for HPLC analysis. The C18 reversed-phase analytical column (250 mm × 4.6 mm, 5 μm, Greenherbs Science and Technology, Beijing, China) was maintained at 25°C, with 0.1% formic acid (A) and acetonitrile (B) as the mobile phase with a flow rate of 0.8 mL/min. The gradient elution conditions of the mobile phase B were: 0–12 min, 2–8%; 12–15 min, 8–13%; 15–30 min, 13–18%; 30–50 min, 18–30%; 50–60 min, 30–50%; 60–70 min, 50–70%; 70–80 min, 70–90%; 80–85 min, 90–100%; 85–90 min, 100–2%. The DAD was set in four wavelengths: 280 nm for identification of gallic acid, (+)-catechin, epicatechin, rosmarinic acid, baicalin, luteolin, apigenin, hesperetin, and baicalein; 310 nm for chlorogenic acid; 340 nm for ferulic acid; and 360 nm for rutin. Finally, compounds were identified and quantified by comparison with the retention times and peak areas from standards, and information on these compounds is listed in Table 1.

**TABLE 1:** List of compounds identified by HPLC.

Number	Retention time (min)	Name	Detection wavelength (nm)
1	9.99	Gallic acid	280
2	16.78	(+)-Catechin	280
3	22.4	Chlorogenic acid	310
4	23.46	Epicatechin	280
5	26.936	Rutin	360
6	29.743	Ferulic acid	340
7	39.83	Rosmarinic acid	280
8	43.86	Baicalin	280
9	46.442	Luteolin	280
10	56.102	Apigenin	280
11	56.87	Hesperetin	280
12	62.4	Baicalein	280

### Metabolomics Analysis

The metabolomics analysis was performed by Shanghai Applied Protein Technology Co. Ltd. An appropriate sample was added to precooled methanol/acetonitrile/aqueous solution (2:2:1, V/V) and vortex-mixed. After the ultrasound for 30 min at 4°C, the mixed sample was kept at -20°C for 10 min and then centrifuged (14,000 g, 4°C) for 20 min. The supernatant was vacuum-dried and redissolved in 100 μL acetonitrile solution (acetonitrile: water = 1:1, V/V), and then centrifuged again at 14,000 g for 15 min at 4 °C. Finally, the supernatant was eventually used for mass spectrometry (MS) analysis.

The samples were separated by an ultra-high performance liquid chromatography (UHPLC) system (Agilent, Santa Clara, United States) with a C18 column (1.7 μm, 2.1 mm × 100 mm). The sample was injected using an autosampler and the injection volume was 2 μL. The flow rate was 0.40 mL/min and the column temperature was 40°C. The mobile phase comprised of eluent A (water with 25 mM ammonium acetate and 0.5% formic acid) and eluent B (methanol). The gradient elution program was set as follows: 0–0.5 min, 5% B; 0.5–10 min, 5–100% B; 10–12 min, 100% B; 12.0–12.1 min, 100–5% B; and a final 12.1–16 min, 5% B.

MS/MS was conducted in both positive and negative ion modes using electrospray ionization (ESI) on AB Triple TOF 6600 (AB Sciex, United States). The ESI source condition was set as follows: Ion Source Gas1 (60 psi), Ion Source Gas2 (60 psi), Curtain gas (30 psi), Source temperature (600°C), Ion Sapary Voltage Floating (± 5,500 V), TOF MS scan m/z range (60–1,000 Da), Declustering potential (± 60 V), and Collision energy (35 ± 15 eV). The information-dependent acquisition (IDA) conditions were: exclude isotopes within 4 Da, and candidate ions to monitor per cycle was 10. All samples were injected in sequence. The quality control (QC) samples were pooled samples prepared from mixed aliquots of equal volume from all samples to validate system stability and repeatability.

### Statistical Analyses

All experiments were done in triplicate and expressed as mean ± standard deviation. The SPSS 22 software package was used to perform a one-way ANOVA for the determination of statistical significance. Principal component analysis (PCA), Spearmen correlation, volcano plots, heatmap, and associated network diagram were generated with R software (version 4.0.6). Significantly regulated metabolites between groups were determined by Log_2_FC ≥ 1.5, *p* < 0.05.

## Results

### pH Values

The variation in pH values of the fermentation broth reflects the degree of fermentation (23). Table 2 indicated that the pH values presented a dramatic decrease in the early stage, after which the trend became flat. There was a significant difference after a 1-day fermentation for each group when compared to the unfermented sample (*p* < 0.05). Among them, ATCC 53013 and CICC 6038 decreased faster than other probiotics from the third day (*p* < 0.05), which implied they exhibited the strongest fermentation properties. Overall, the CN control groups also showed similar trends to the experimental groups, but finally decreased to a greater extent. This indicated that the addition of CN sources might also contribute to increasing the extent of fermentation.

**TABLE 2:** Change in the pH, TPC, and TFC of FEPL during different fermentation stages for different probiotics.

	Probiotics	Days							
		
		0	1	2	3	4	5	6	7
pH	SWFU D16	5.43 ± 0.01*^a^*	4.31 ± 0.01^b^	4.22 ± 0.01^b^	4.32 ± 0.23^b^	4.17 ± 0.01^b^	4.24 ± 0.03^b^	4.22 ± 0.03^b^	4.22 ± 0.02^b^
	SWFU D16 + CN	5.63 ± 0.02^a^	3.90 ± 0.01^b^	3.68 ± 0.01^c^	3.55 ± 0.01^d^	3.50 ± 0.02^e^	3.44 ± 0.01*^f^*	3.40 ± 0.02^g^	3.41 ± 0.03^g^
	ATCC 8014	5.92 ± 0.02^a^	5.44 ± 0.02^b^	4.67 ± 0.01^c^	4.31 ± 0.01^d^	4.15 ± 0.03^e^	3.93 ± 0.03^f^	3.81 ± 0.01^g^	3.77 ± 0.02*^h^*
	ATCC 8014 + CN	5.77 ± 0.12^a^	5.48 ± 0.03^b^	4.80 ± 0.27^c^	4.52 ± 0.08^d^	4.45 ± 0.01^e^	4.43 ± 0.02^e^	4.45 ± 0.02^e^	4.46 ± 0.02^e^
	ATCC 53013	5.49 ± 0.03^b^	5.53 ± 0.01^a^	5.44 ± 0.01^c^	4.48 ± 0.01^d,e^	4.45 ± 0.01^f^	4.45 ± 0.03*^e,f^*	4.46 ± 0.01^e,f^	4.50 ± 0.01^d^
	ATCC 53013 + CN	5.71 ± 0.02^a^	5.70 ± 0.01^a^	5.40 ± 0.01^b^	3.76 ± 0.02^c^	3.59 ± 0.01^d^	3.45 ± 0.02^e^	3.40 ± 0.01^f^	3.33 ± 0.01^g^
	CICC 6038	5.70 ± 0.03^a^	5.58 ± 0.02^b^	5.50 ± 0.02^c^	5.62 ± 0.01^b^	4.48 ± 0.07^e^	4.55 ± 0.02^d^	4.55 ± 0.01^d^	4.56 ± 0.01^d^
	CICC 6038 + CN	5.71 ± 0.03^a^	5.65 ± 0.03^a^	5.71 ± 0.01^a^	5.29 ± 0.01^b^	3.83 ± 0.02^c^	3.49 ± 0.13^d^	3.48 ± 0.02^d^	3.49 ± 0.01^d^
	ATCC 334	5.67 ± 0.02^c^	5.73 ± 0.01^b^	5.75 ± 0.01^a^	5.37 ± 0.01^d^	4.74 ± 0.01^e^	4.66 ± 0.01^g^	4.69 ± 0.01^f^	4.67 ± 0.01^g^
	ATCC 334 + CN	5.68 ± 0.04^a^	5.80 ± 0.02^b^	4.47 ± 0.02^c^	3.78 ± 0.01^d^	3.60 ± 0.02^e^	3.53 ± 0.01^f^	3.43 ± 0.04^g^	3.42 ± 0.02^g^
	CICC 6045	5.69 ± 0.02^d^	5.65 ± 0.01^e^	5.71 ± 0.03^c,d^	5.77 ± 0.02^a^	5.73 ± 0.01^b,c^	5.72 ± 0.03^c,d^	5.73 ± 0.00^b,c^	5.76 ± 0.01*^a,b^*
	CICC 6045 + CN	5.62 ± 0.01^c^	5.70 ± 0.01^b^	5.73 ± 0.01^a^	3.95 ± 0.01^d^	3.80 ± 0.02^e^	3.50 ± 0.01^f^	3.42 ± 0.01^g^	3.37 ± 0.01*^h^*
TPC (%)	SWFU D16	100.00 ± 1.11^a^	94.12 ± 3.53^b^	87.77 ± 1.39^c^	91.68 ± 3.40^b,c^	66.63 ± 2.45^e^	69.04 ± 3.12^e^	76.90 ± 1.73^d^	53.10 ± 1.38^f^
	SWFU D16 + CN	100.22 ± 2.22^a^	95.72 ± 1.85^a^	80.87 ± 1.44^b^	66.56 ± 3.83^c^	65.49 ± 3.56^c^	58.25 ± 3.12^d^	44.54 ± 1.73^e^	39.56 ± 1.72^e^
	ATCC 8014	100.00 ± 0.49^b,c^	78.60 ± 2.64^d^	85.24 ± 5.05^d^	113.07 ± 5.19^a^	100.16 ± 3.46^b,c^	96.71 ± 6.03^c^	107.23 ± 7.25*^a,b^*	57.63 ± 1.25^e^
	ATCC 8014 + CN	100.00 ± 5.45^b,c^	110.34 ± 1.96^a^	84.58 ± 2.87^d^	89.35 ± 4.86^d^	101.78 ± 8.59*^a,b^*	51.00 ± 1.19^e^	92.45 ± 0.83^c,d^	86.07 ± 2.67^d^
	ATCC 53013	100.00 ± 1.61*^a,b^*	95.64 ± 5.50*^a,b^*	105.44 ± 9.35^a^	99.90 ± 3.87*^a,b^*	85.07 ± 5.85^c,d^	82.84 ± 2.44^c,d^	78.04 ± 3.28^d^	91.25 ± 4.22^b,c^
	ATCC 53013 + CN	100.00 ± 4.60^a^	105.48 ± 1.53^a^	91.06 ± 5.56^b^	70.17 ± 3.48^c^	68.77 ± 0.84^c^	70.11 ± 2.21^c^	60.60 ± 1.48^d^	69.89 ± 1.85^c^
	CICC 6038	100.00 ± 2.40^c^	146.27 ± 8.70^a^	105.63 ± 4.34^c^	115.75 ± 2.47^b^	91.18 ± 3.72^d^	57.53 ± 3.85^e^	57.39 ± 1.05^e^	64.02 ± 1.83^e^
	CICC 6038 + CN	100.00 ± 2.53^c^	126.83 ± 2.46^b^	132.78 ± 2.94^a^	99.63 ± 2.48^c^	98.07 ± 3.88^c^	83.25 ± 3.30^d^	83.46 ± 1.18^d^	76.39 ± 1.46^e^
	ATCC 334	100.00 ± 8.24^c,d^	97.47 ± 0.64^c,d^	90.47 ± 9.17^d^	118.97 ± 4.89^a^	64.78 ± 5.04^f^	113.83 ± 1.35*^a,b^*	105.90 ± 1.77^b,c^	102.32 ± 5.50^c^
	ATCC 334 + CN	100.00 ± 1.86^a^	96.75 ± 2.99^a^	68.85 ± 2.57^b^	41.77 ± 2.92^f^	53.82 ± 2.55^d^	49.04 ± 0.99^e^	61.65 ± 1.05^c^	63.16 ± 1.78^c^
	CICC 6045	100.00 ± 4.88*^a,b,d^*	93.78 ± 4.94^b,c^	99.27 ± 0.34*^a,b^*	91.80 ± 2.46^c,d^	87.14 ± 3.95^d^	95.03 ± 4.60^b,c^	49.42 ± 1.81^e^	105.72 ± 0.65^a^
	CICC 6045 + CN	100.00 ± 0.98^a^	66.01 ± 3.97^d^	60.39 ± 3.18^e^	81.80 ± 1.01^b^	75.00 ± 0.73^c^	43.47 ± 1.34*^f,g^*	41.16 ± 1.34^g^	45.61 ± 0.63^f^
TFC (%)	SWFU D16	100.00 ± 2.93^a^	73.27 ± 1.91^b^	65.34 ± 4.09^c^	71.30 ± 3.83^b^	50.84 ± 2.02^d^	48.04 ± 3.39^d^	60.88 ± 1.58^c^	16.39 ± 1.53^e^
	SWFU D16 + CN	100.00 ± 2.93^a^	75.14 ± 9.09^b^	68.95 ± 7.26^b^	49.33 ± 3.93^c^	36.43 ± 3.63^d^	35.45 ± 2.65^d,e^	26.01 ± 3.66^e,f^	17.19 ± 0.21^f^
	ATCC 8014	100.00 ± 10.75^c,d^	89.17 ± 6.30^d^	144.86 ± 4.99^a^	130.39 ± 17.41*^a,b^*	101.89 ± 6.03^c,d^	100.68 ± 7.39^c,d^	116.90 ± 3.14^b,c^	44.51 ± 6.26^e^
	ATCC 8014 + CN	100.00 ± 2.80^a^	97.78 ± 4.95^a^	72.36 ± 5.77^b^	53.45 ± 5.33^c^	52.00 ± 3.89^c^	3.14 ± 0.45^e^	30.52 ± 3.78^d^	25.22 ± 0.31^d^
	ATCC 53013	100.00 ± 4.45^c^	152.38 ± 4.75^a^	150.00 ± 6.20^a^	131.87 ± 10.76^b^	50.00 ± 2.23^d^	112.51 ± 10.26^c^	107.35 ± 13.92^c^	110.61 ± 4.17^c^
	ATCC 53013 + CN	100.00 ± 2.78*^a,b^*	107.39 ± 13.99^a^	93.22 ± 9.81^b^	34.84 ± 6.11^c^	23.74 ± 3.99^c,d^	15.01 ± 1.20^d^	20.09 ± 2.81^d^	16.82 ± 1.79^d^
	CICC 6038	100.00 ± 9.54^b,c^	126.57 ± 2.72^a^	138.09 ± 8.69^a^	126.63 ± 8.37^a^	85.75 ± 13.71^c,d^	70.06 ± 2.30^d^	90.23 ± 3.87^b,c^	102.35 ± 7.72^b^
	CICC 6038 + CN	100.00 ± 10.84^b^	122.15 ± 3.24^a^	137.68 ± 5.45^a^	81.18 ± 17.03^c^	50.32 ± 3.95^d^	18.33 ± 0.81^e^	17.61 ± 2.60^e^	17.50 ± 1.52^e^
	ATCC 334	100.00 ± 0.62^a^	95.20 ± 2.91^a^	66.84 ± 2.15^c^	83.48 ± 4.40^b^	34.27 ± 1.84^d^	84.50 ± 5.24^b^	83.11 ± 1.88^b^	70.27 ± 5.09^c^
	ATCC 334 + CN	100.00 ± 13.73^a^	93.23 ± 5.36^a^	51.26 ± 6.77^b^	6.47 ± 1.71^c^	17.66 ± 1.55^c^	10.28 ± 1.79^c^	12.04 ± 0.88^c^	13.33 ± 0.82^c^
	CICC 6045	100.00 ± 4.62^a^	85.17 ± 6.87^b,c^	88.43 ± 4.85^b^	75.98 ± 4.20^c,d^	102.78 ± 3.18^a^	63.01 ± 4.35^e^	37.58 ± 4.11^f^	69.80 ± 5.73^d,e^
	CICC 6045 + CN	100.00 ± 6.39^a^	86.42 ± 4.36^b^	95.15 ± 0.43^a^	86.76 ± 1.96^b^	99.88 ± 2.12^a^	19.45 ± 1.43^c^	16.70 ± 2.40^c^	17.15 ± 1.81^c^

*Different lower-case letters in the same row indicate significant differences at p < 0.05.*

### Total Phenolic and Flavonoid Content

From Table 2, the TPC and TFC of FPFL behaved in a general trend of increasing first and then decreasing for almost all probiotics besides SWFU-D16, while the turning points were pooled from days 1–3. Overall, we observed substantial decreases in TPC and TFC after fermentation (*p* < 0.05). Similarly, each of the corresponding CN control groups displayed a significantly higher reduction than that of the probiotic group (*p* < 0.05). Although final losses were significant for TPC and TFC, a significant improvement was observed in short-term fermentation (1–3 days), which was consistent with the previously reported literature (24, 25). Among them, the maximum TPC increase was observed in CICC 6038 (146.27% at 1 day), while the largest TFC increase was found for ATCC 53013 (152.38% at 2 days). A possible reason for the increase of TPC and TFC in the early fermentation stage was the vigorous growth of microorganisms, and their high metabolic activities consumed the components in PFL, such as starch, protein, pectin, etc., which led to the release of phenolics and flavonoids (26). With the continuation of the fermentation process, the substances in PFL were utilized, degraded, and transformed by microorganisms, which is the cause of the decrease in TPC and TFC (27).

### Antioxidant Abilities

The antioxidant ability of the sample depends on the presence of various compounds with different action mechanisms. In this study, three methods were performed to evaluate the antioxidant effects of FPFL, including DPPH, ABTS radical scavenging abilities, and FRAP. Of these, DPPH and ABTS radical scavenging assays utilize hydrogen atom transfer and single electron transfer reaction mechanisms, while the FRAP assay takes up the single electron transfer method (28, 29).

**FIGURE 1:**
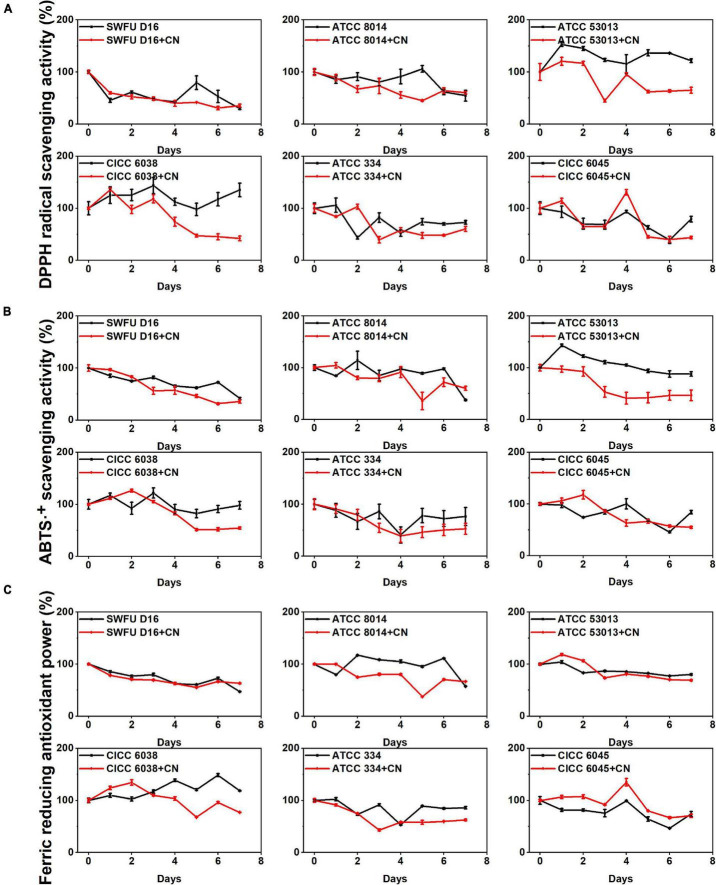
Changes in the antioxidant abilities of FPFL during the fermentation process for different probiotics. **(A)** DPPH⋅ scavenging ability. **(B)** ABTS⋅^+^ scavenging ability. **(C)** FRAP. Asterisks (*) represent the significant difference when comparing the unfermented sample (0 days) (*p* < 0.05*), the samples from the same group are represented by the same color.

From Figure 1, all the three antioxidant abilities were decreased after 7 days of fermentation (*p* < 0.05), and this decrease was more pronounced in the CN control groups. During short-term fermentation time, the antioxidant abilities were substantially improved for ATCC 53013, CICC 6038, and ATCC 8014 (*p* < 0.05), regardless of whether they were in the CN control groups or the unfermented samples. Similar to TPC and TFC, the time points with the highest antioxidant ability were focused primarily on the first 1–3 days. For DPPH and ABTS radical scavenging ability, the highest increase was noted for ATCC 53013 at 1 day (153.03 and 143.26%), while for FRAP, it was 148.63% (CICC 6038 at 6 days). It was also observed that FRAP was always high from the beginning of fermentation to the end of fermentation (1–7 days), which was different from DPPH and ABTS radical scavenging abilities, and this also illustrated the different antioxidant mechanisms between them. Overall, FPFL demonstrated excellent antioxidant abilities after short-term fermentation, of which CICC 6038 and ATCC 53013 had the strongest boosting effects, which was consistent with a previous study reported by Ru et al. (30).

**FIGURE 2:**
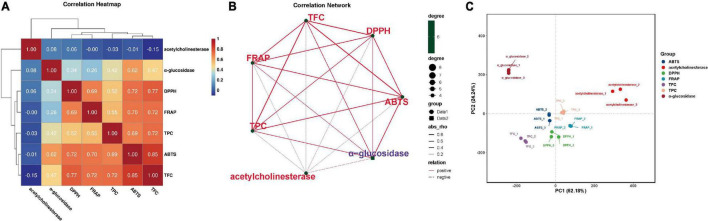
Associations among different indicators. **(A)** Correlation heatmap. **(B)** Correlation network. **(C)** Principal component analysis. TPC, total phenolic content; TFC, total flavonoid content; DPPH, DPPH⋅scavenging activity; ABTS, ABTS⋅+ scavenging ability; FRAP, ferric reducing antioxidant power; α-glucosidase, α-glucosidase inhibition ability; AChE, acetylcholinesterase inhibition ability.

A previous study reported that short-term fermentation could release bound phenols in plants, which in turn increased the TPC and TFC, and improved the antioxidant ability (31). To elucidate the similarities and differences between TPC, TFC, antioxidant ability, and the other variables more clearly, the correlation analysis and principal component analysis (PCA) were further performed and are displayed in Figure 2 in the form of the correlation heatmap, correlation network, and PCA graph, respectively. The high and significant correlation coefficients indicated higher correlations between TPC/TFC and different antioxidant abilities, which had also been proved by the cluster distances, the intersection networks, and the relative distance in 2D principal-component space. This implicated phenolics and flavonoids, especially, phenolics were the main factors contributing to antioxidant ability, which also explained the similar changing trends among TPC/TFC and antioxidant abilities.

### α-Glucosidase Inhibition Ability

**FIGURE 3:**
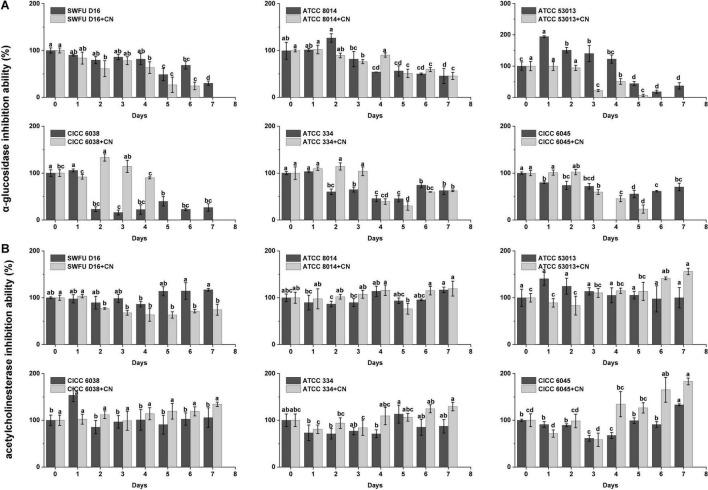
The enzymes’ inhibition abilities of the FPFL during the fermentation process for different probiotics. **(A)** α-glucosidase inhibition ability. **(B)** Acetylcholinesterase inhibition ability. Mean ± standard deviation, (*n* = 3). Bars with different letters indicate a significant difference (*p* < 0.05).

α-Glucosidase is an essential target for type II diabetes. Inhibition of α-glucosidase can delay carbohydrate digestion and glucose absorption, which in turn leads to reducing postprandial blood glucose levels, and ultimately improves mitigation of diabetes and its complications (32). From Figure 3A, most of the probiotics could enhance the α-glucosidase inhibition ability of FPFL during the first 1–3 days (*p* < 0.05). Among them, ATCC 53013 showed the highest level of increase after fermentation for 1 day, 194.14% as large as the unfermented sample, and maintained at a high level (> 122%) over the following 3 days. Followed by ATCC 8014 (126.61% at the 2 days), in brief, these two strains were significantly better compared to their corresponding CN control groups. The reason may be that the addition of CN could facilitate PFL fermentation, which results in a higher degree of conversion and degradation of the active ingredients in FPFL, which in turn, reduces its α-glucosidase inhibition ability. However, the CN control groups of ATCC 334 and CICC 6045 presented better α-glucosidase inhibition ability than without CN samples, likely because different probiotics had different lactic fermentation, acid tolerance, and CN source utilization abilities (33). The proper amount of CN addition can make the bioactive ingredients of fermentation substrate release most, which in turn gives a stronger α-glucosidase inhibition ability. For example, *L. bulgaricus*, *L. casei*, *L. fermentum*, *L. delbrueckii*, and *L. lactis* showed different lactic acid yields under the same CN conditions, while lactic acid yields instead decreased at higher concentrations of carbon source for *L. casei* (34, 35). In addition, *Bacillus licheniformis* is a non-lactic acid bacteria regarded as a probiotic. It was found that high CN source concentration stimulated the cell growth of *B. licheniformis* but reduced its trypsin activity, while the opposite result was observed with low concentrations (36). From significant but lower correlations, and the long distances in the PCA graph (Figure 2), while the α-glucosidase inhibition ability was significantly influenced by TPC, TFC, and other antioxidant material, it was not determined entirely by them. Overall, the α-glucosidase inhibition ability of FPFL was decided by the integrated effect of various bioactive ingredients, and ATCC 53013 proved to be an excellent probiotic tool to increase the α-glucosidase inhibition ability (30).

### Acetylcholinesterase Inhibition Ability

Up to today, acetylcholinesterase (AChE) is an attractive target for the treatment of Alzheimer’s disease, and acetylcholinesterase inhibitors represent the major approved drugs to treat this neurodegenerative disease (37). With the advantage of being safe, cost-effective, and efficacious, natural food or medicinal plants are considered novel strategies for the prevention and treatment of Alzheimer’s disease. Gratifyingly, almost all the samples presented a certain inhibition activity against AChE, and all six probiotics could enhance the AChE inhibition ability of FPFL after fermentation (Figure 3B), which is consistent with the conclusion of Li (38). Of these, after fermentation for 1 day, ATCC 53013 and CICC 6038 showed the strongest upregulation with the maximum value of 140.48 and 153.39%, respectively. Surprisingly, most of the CN control groups showed stronger AChE inhibition ability except SWFU D16. The reason could be that SWFU D16 was isolated from goat milk cake, which was not fit for PFL fermentation, and there was no obvious effect of CN-addition. Considering that the addition of CN had a greater degree of conversion and degradation for FPFL, we supposed that these new products were more likely to have a role in the inhibition of AChE. The correlation analysis and PCA analysis further validated our results that AChE inhibition ability was also weakly correlated with TPC/TFC, antioxidant abilities, and α-glucosidase inhibition ability, and falls away from them on the PCA plot.

### High-Performance Liquid Chromatography Analysis Results

**FIGURE 4:**
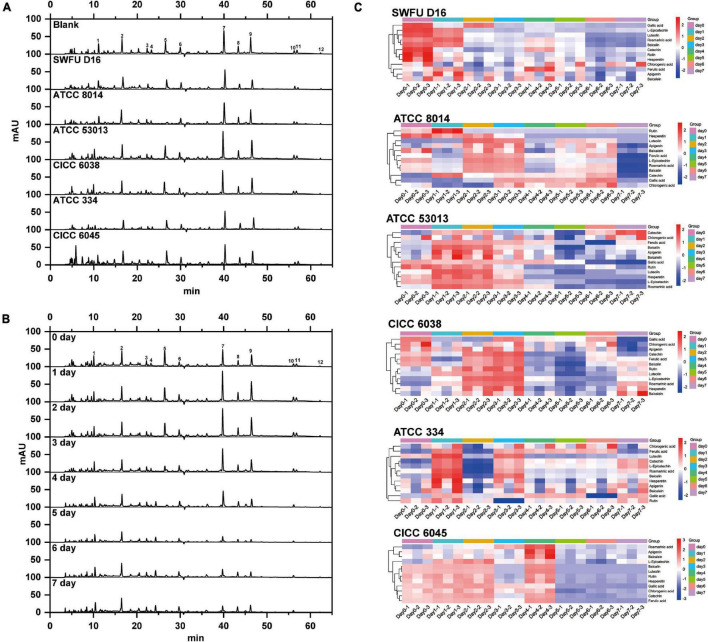
HPLC chromatogram and heatmap of FPFL. **(A)** Chromatogram of FPFL for different probiotics at 280 nm on 1 day of fermentation. **(B)** Chromatogram of FPFL during different fermentation periods for ATCC 53013 at 280 nm. **(C)** Heatmap of compounds content of FPFL during different fermentation periods and different probiotics.

To elucidate the changes of the various compounds in PFL fermented by different probiotics at different fermentation stages, HPLC analysis was performed to present their chromatographic characterizations. According to the studies published previously by us and others, a total of 12 compounds were confirmed and quantified by comparing their retention time and UV spectrum to the standard, including gallic acid, (+)-catechin, chlorogenic acid, epicatechin, rutin, ferulic acid, rosmarinic acid, baicalin, luteolin, apigenin, hesperetin, and baicalein (12, 17, 39). These compounds are labeled in Figures 4A,B with Arabic numerals according to their elution order.

Firstly, samples fermented by different probiotics for 1 day were selected for comparative analysis, and their chromatograms at 280 nm were shown in Figure 4A. For all probiotics, their chromatograms were essentially identical, while there was some difference in the height and area of some chromatographic peaks. Among them, the chromatographic peak of rosmarinic acid (peak 7) of ATCC 53013 was significantly higher than other probiotics. Similarly, the same phenomena can also be observed in other peaks for different probiotics. When compared to the blank sample (without probiotics), CICC 6045 had significantly higher and more chromatographic peaks in the first 10 min, which indicated that it could produce more high-polarity compounds.

Among all six probiotics, ATCC 53013 showed excellent promoting effects on nearly all the evaluation metrics of PFL, especially for α-glucosidase inhibition ability, thus it was a superior probiotic for the fermentation of PFL. In accordance with these experiment results, we choose FPFL fermented by ATCC 53013 to compare the changes of the various compounds each day. As shown in the chromatographs in Figure 4B, the peaks of rutin, hesperetin, and baicalein (peaks 5, 11, 12) decreased gradually and almost disappeared on day 4, while peaks of rosmarinic acid, baicalin, luteolin, and apigenin (peaks 7, 8, 9, 10) were increased first and decreased afterward, reaching the highest levels on the first day. For other peaks, there were no obvious changes. Interestingly, these compounds with large content changes were almost all flavonoids, except for rosmarinic acid, which means the biotransformation of flavonoids might be one of the major reactions during fermentation. Of which, luteolin (peak 9) has been previously reported to possess beneficial biological activities, such as anti-inflammatory, anti-allergic, and antibacterial activities, and could stably bind to α-glucosidase *via* hydrophobic force, which in turn caused inactivation of α-glucosidase (40). Similarly, apigenin (peak 10) had potential therapeutic effects on a variety of neurodegenerative diseases such as Alzheimer’s disease and Parkinson’s disease (41). In addition, rosmarinic acid (peak 7) also had a lot of interesting pharmacological activities, including antiviral, antibacterial, anti-inflammatory, and antioxidant abilities, and was identified as a potential treatment for Alzheimer’s disease (42, 43). The above results explained the improvement of antioxidant abilities, α-glucosidase inhibition ability, and acetylcholinesterase inhibition ability of PFL after fermentation.

To better present the changes of different probiotic groups at different stages of fermentation, the contents of these 12 compounds were visually exhibited as a heatmap in Figure 4C. Blue represented low content while red represented high content. It could be clearly seen that SWFU D16 and CICC 6045 showed an obvious trend from red to blue, which indicated the decreasing trend of the content of these compounds in these two probiotic groups. For ATCC 8014, ATCC 53013, CICC 6038, and ATCC 334 groups, their colors changed from blue to red and then to blue, showing that the levels of these compounds were first increased and then decreased. In addition, when compared with other probiotic groups, ATCC 53013, ATCC 8014, and CICC 6038 groups had significantly more red areas and darker color groups within 3 days of fermentation, which implied that the increase of these major compounds was more accentuated in these probiotic groups. Again, these results strongly supported the preceding results, especially for ATCC 53013 in Figure 4B. The significant difference between different strains could not only be found according to the heatmap but also between the different compounds from the same probiotic group. For the rutin, rosmarinic acid, and luteolin of these compounds, clear red color in the first 3 days and distinct changes were clearly observed. They have been reported to be the major compounds of PFL in our previous reports and exhibit excellent biological activity (12, 17).

Taken together, the above results suggested that probiotic fermentation had different effects on various compounds in PFL, and different probiotics exerted different effects, which might be due to the various mechanisms of action of probiotic fermentation, including formation, transformation, and degradation.

### Metabolomic Analysis

**FIGURE 5:**
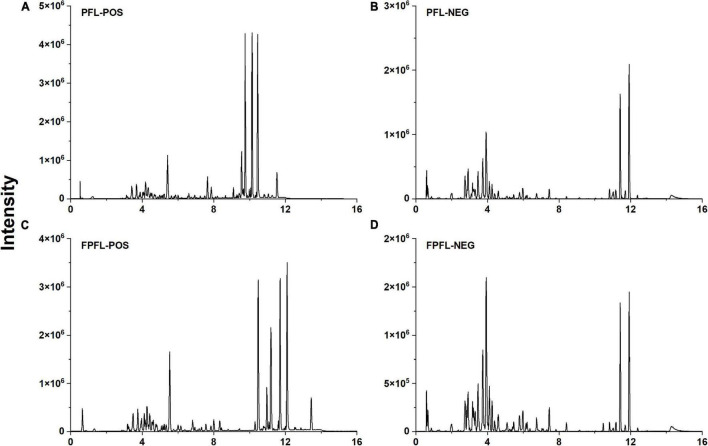
Representative base peak chromatograms (BPC) of PFL and FPFL in the positive **(A,C)** and negative **(B,D)** ions mode.

To further elucidate the metabolite changes during fermentation, we analyzed the metabolites of FPFL (fermented by ATCC 53013 for 1 day) using a non-targeted metabolomics approach based on UHPLC–Triple–TOF–MS/MS. It is generally believed that ESI positive ion mode is more sensitive than the negative ion mode, while the negative ion mode is more suitable for acidic compounds (44). Positive and negative ion modes were complementary and they were both therefore used for analysis. The representative base peak chromatograms (BPC) of PFL (unfermented sample) and FPFL in both modes are shown in Figures 5A–D, respectively. According to the BPC plots, there were significant differences in the metabolites of the samples before and after fermentation. The spectral signal intensities of FPFL were weaker than PFL, which indicated that fermentation reduced the content of the primary metabolites and secondary metabolites in PFL.

**FIGURE 6:**
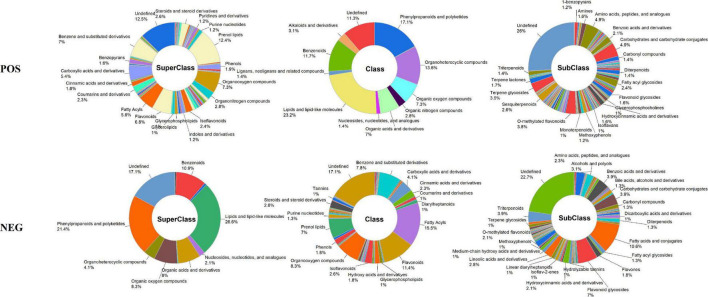
The schematic diagram of the different classifications of the metabolites of FPFL.

The metabolites were identified by comparing accurate mass (error < 10 ppm), retention times, fragmentation patterns, and collision energies against standard compounds and the in-house database (Shanghai Applied Protein Technology Co., Ltd, Shanghai, China), and their confidence levels of compound annotations were at level 2 or higher (45). In this study, a total of 961 metabolites were identified, among which 574 and 387 were detected in positive mode and negative mode (Supplementary Table 1), respectively. To show the composition and classification of these metabolites more intuitively, the corresponding pie diagrams are plotted in Figure 6. The different colors in each pie chart represented different classifications, and the area represented the relative proportion of metabolites in the classification. As exhibited in Figure 6, the predominant superclass metabolites were lipids and lipid-like molecules, phenylpropanoids and polyketides, organoheterocyclic compounds, benzenoids, and organic oxygen compounds. At the class level, they were mainly prenol lipids, fatty acyls, flavonoids, organooxygen compounds, as well as benzene, and substituted derivatives. While at the subclass level, they were fatty acids and conjugates, flavonoid glycosides, carbohydrates and carbohydrate conjugates, o-methylated flavonoids, and terpene glycosides.

**FIGURE 7:**
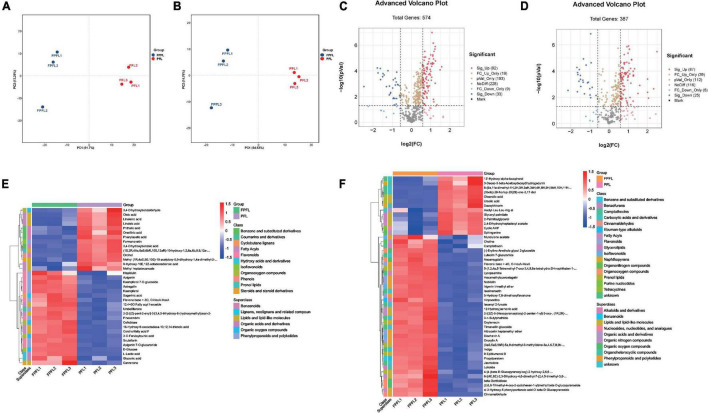
PCA, volcano plots, and heat map show the changes of metabolites in PFL and FPFL. **(A)** Score plot in positive ions mode, **(B)** score plot in negative ions mode. Represent high cohesion within groups and good separation among before and after fermentation. **(C)** Volcano plot in positive ions mode. **(D)** Volcano plot in negative ions mode. The blue dots represent significantly downregulated differentially expressed metabolites, the red dots represent significantly upregulated differentially expressed metabolites. Significantly different metabolites between groups were determined by *p* < 0.05 and an absolute Log_2_FC (fold change) ≥ 1. **(E)** Heat map in positive ions mode, **(F)** heat map in negative ions mode. Each sample is represented by one column, and each metabolite is visualized in one row. Red indicates high abundance; blue indicates relatively low metabolite abundance.

Next, we sought to quantify differences in metabolite compositions before and after fermentation with principal components analysis (PCA). In Figures 7A,B, highly significant discrimination between two groups, both in positive and negative ion modes, is shown, indicating significant variation in metabolites of PFL after fermentation. To display differentially metabolites, the volcano plots (Figures 7C,D) were constructed based on statistical value *p* < 0.05 and fold change ≥ 1.5. The results showed that 92 metabolites were significantly up-regulated and 33 were significantly down-regulated in positive ion mode, while 87 metabolites were significantly up-regulated and 25 were significantly down-regulated in negative ion mode. According to the variable importance in projection (VIP) value obtained by OPLS-DA and the results of *t*-tests, metabolites that had both VIP > 1 and *p* < 0.05 were selected to generate hierarchical cluster heatmaps (Figures 7E,F). The heatmaps in both positive and negative ion modes revealed significant changes in these compounds in different classes, including primary metabolites (fatty acids, lipids, and nucleosides) and secondary metabolites (alkaloids, flavonoids, phenols, and terpenoids). In brief, the upregulated metabolites were more numerous compared with the downregulated metabolites after fermentation, and the negative ion mode provided a higher ability to detect more significant metabolites.

**FIGURE 8:**
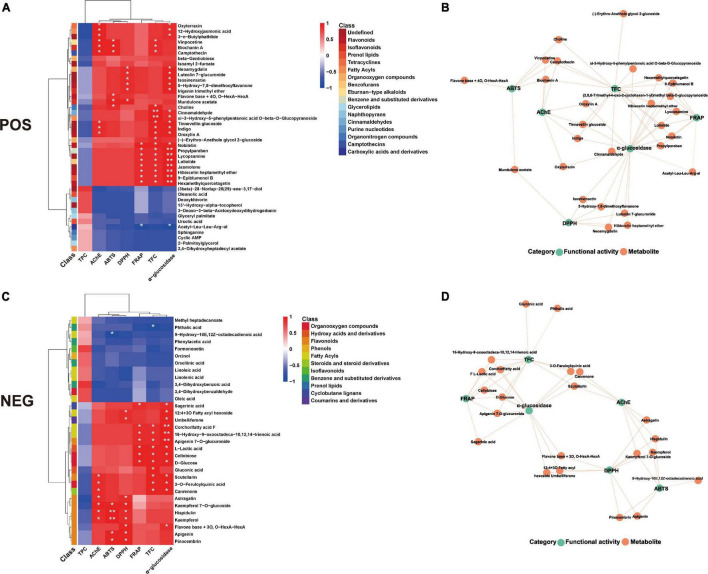
Spearman’s analysis and associated network diagram show the correlation between metabolites and 6 functional activities. **(A,C)** Spearman’s analysis of FPFL in the positive and negative ions mode. **(B,D)** Associated network of FPFL in positive and negative ions mode. Asterisks represent *p < 0.05, **p ≤ 0.01, respectively.

To further explore the correlation between metabolites and functional activity, a series of Spearman’s correlation analyses were conducted and illustrated with heatmaps and network diagrams in Figure 8. Interestingly, most of the metabolites that highly correlated with the previously described activity indicators belonged to the upregulated metabolites, no matter in both positive and negative ion modes. α-Glucosidase inhibition ability and TFC were significantly associated with the greatest number of metabolites, followed by three antioxidant abilities, and acetylcholinesterase inhibition ability, which further explained the significant improvements of these metrics, especially the α-glucosidase inhibition ability. An unexpected observation was that no metabolites were significantly associated with TPC, which was also in agreement with the results in Table 2, that no significant changes were observed in TPC after 1 day of fermentation. The complex relationships between these indicators and key metabolites were further illustrated in Figures 8B,D. In addition, it was found that classifications at the class level of these key metabolites majorly belonged to flavonoids, isoflavonoids, prenol lipids, organooxygen compounds, hydroxy acids, and derivatives, phenols, and fatty acyls. This illustrated the complicated chemical and biological changes that occurred during the fermentation process involving almost all the constituents of PFL.

It is well known that the secondary metabolites of plants are mainly regulated by their metabolic pathways, which in turn affect their functional activities. The KEGG pathway database^1^ is a main public database of metabolic pathways, which could be used in studies of gene expression information and metabolite accumulation in a general network. In this study, all the differential metabolites were entered into the KEGG database for pathway analysis and obtained the pathway information of metabolite participation. Pathway enrichment analysis was performed using the KEGG ID of differential metabolites to derive the metabolic pathway enrichment results.

**FIGURE 9:**
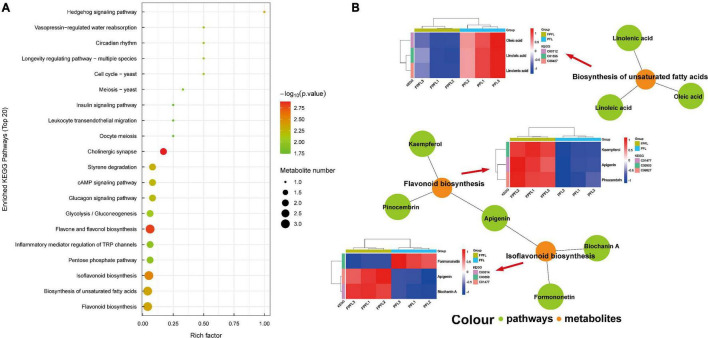
Enriched KEGG pathways based on significantly different metabolites between PFL and FPFL. **(A)** Pathway enrichment analysis. Each bubble in the bubble graph represents a metabolic pathway, the bubble size being proportional to the enrichment. The top 20 items with the highest *p* values were selected. **(B)** Associated network diagram. The elliptical nodes represent pathways, and the rectangular nodes represent metabolites.

A total of 97 signaling pathways were significantly enriched through the KEGG pathway enrichment analysis, and these pathways are listed in Supplementary Table 2. As shown in Figure 9A, the bubble plot demonstrated the Top 20 most strongly enriched pathways, and flavonoid biosynthesis, biosynthesis of unsaturated fatty acids, and isoflavonoid biosynthesis were the most enriched pathways. This validated the results described in the previous paragraphs, with flavonoids and isoflavonoids being the most associated with their corresponding bioactivities. Flavonoids are dominant secondary metabolites in plants and play an important role in their biological functions. Notably, accumulation of antioxidants was often observed in the flavonoid biosynthesis pathway (46). Figure 9B presented the network plot of the relationships between these pathways and differential metabolites, and the metabolic differences of these metabolites were revealed by the corresponding heatmap. Among them, apigenin, kaempferol, and pinocembrin were the differential metabolites involved in the flavonoid biosynthesis pathway, and apigenin, biochanin A, and formononetin were involved in the isoflavonoid biosynthesis pathway, while for unsaturated fatty acid biosynthesis pathway, they were linoleic acid, linolenic acid, and oleic acid, respectively. In summary, the results of KEGG enrichment analyses further revealed the diversity of the metabolic pathways of PFL during the fermentation of probiotics and strongly supported the results previously mentioned.

## Discussion

As a traditional medicinal and edible plant, PFL has been shown to possess many bioactive compounds and health properties and has been widely cultivated as a major crop (47). Thus, both from the human nutrition and food bioprocessing perspective, various food processing techniques could be used to improve its sensory and dietary properties. Probiotic fermentation is one of the safe and effective means to improve the nutritional value, functional activity, and pharmacological efficacy of plants, and therefore, could be useful as an important processing technique for PFL. Our study offers several interesting findings. It illustrates the dynamic changes of the active components and functional activities of PFL during the fermentation process and also provides a superior probiotic (ATCC 53013) for the fermentation of PFL. It also deciphers its mechanism of action.

In this study, PFL was fermented by six different probiotics, and the dynamic changes of its active components and functional activities in the fermentation process were evaluated. The results showed that probiotic fermentation had obvious implications for the chemical components and functional activities of PFL, and it showed an overall trend of rising first and then falling. This might be attributed to the metabolism effect of microorganisms, and similar results were also reported in other studies (27, 48, 49). As is well known, for plants, fruits, or vegetables, their phytochemical concentrations and biological activities during the fermentation process are generally affected by the fermentation substrate, strains, and fermentation time (50). By comparing these samples of different times, different probiotics, and the addition of CN source or not, we found that the optimal fermentation time was 1–3 days, and ATCC 53013 was the best probiotic tool for the fermentation of PFL. After short-term fermentation, FPFL showed excellent biological activities, such as antioxidant abilities (ABTS, DPPH, FRAP), α-glucosidase, and Ache inhibition abilities, especially fermented for 1 day using ATCC 53013. Also, the addition of CN could improve the degree of fermentation.

It is known that microorganisms during the fermentation process could release various enzymes that could disrupt the major components of the plant cell wall, such as cellulose, hemicellulose, pectin, lignin, and protein, and promote the transformation of nutrients and hydrolysis of biological macromolecules, as well as increase the content of bioactive substances (49). On the other hand, organic matter and protein in PFL provide a partial CN source for microbial fermentation. Hence, short-term fermentation was found to increase the antioxidant activity of fermented food by the production of different active compounds (51, 52). As the fermentation process proceeded, the substrate was continuously consumed and the lignin degradation by ligninases generates inhibitory compounds, which inhibit cellulolytic enzymes and fermenting microorganisms, thus having a negative effect on fermentation (53, 54). Xiao et al. (48) also found that some active ingredients of tea such as degalloyl catechins and gallic acid increased in the initial stage of fermentation and decreased after long-term fermentation. This could be the reason why FPFL exhibited better biological activities following short-term fermentation. The above results were of importance for PFL fermentation and its application in food/drug engineering.

The HPLC assays further reveal the different effects between different probiotics by analysis of 12 standard compounds. Among them, rutin, rosmarinic acid, and luteolin were the major compounds with large variations. For different probiotics, ATCC 53013 and CICC 6038 showed better enhancement effects. A quantitative analysis further revealed the dynamic variation of these compounds at different fermentation times. The content of these compounds showed a trend of first decline and then rise, while some other compounds continued to rise or declined, or were relatively constant. In addition, some new peaks appeared for some probiotic groups when compared to the unfermented sample, which might imply that some new substances were produced. These changes suggested the different modes of action of probiotic fermentation, including formation, transformation, and degradation, which might be the reason for the variable change of the active components and functional activities of FPFL (55).

The metabolic products of plants were diverse, and many of them were biologically active molecules. Microbial fermentation could alter these metabolites and produce new metabolites, which in turn affect its functional activity. To analyze the metabolic profile changes of PFL before and after fermentation, the UPLC-MS/MS-based metabolomics method was applied. From the informative metabolic profiling data, we found significant differences in metabolites between PFL and FPFL, and they were mainly focused on some primary metabolites and secondary metabolites. Surprisingly, we observed significant upregulation of some flavonoids, phenols, and fatty acids, such as hispidulin, apigenin, kaempferol 7-O-glucoside, astragalin, kaempferol, sagerinic acid, mundulone acetate, choline, camptothecin, etc. Due to most of these compounds showing good functional activities, these could be the main reason for FPFL exhibiting good activity after short-term fermentation (56–58).

Further, KEGG pathway analysis of significantly differential metabolites found 97 metabolic signaling pathways, of which biosynthesis of unsaturated fatty acids, flavonoid biosynthesis, and isoflavonoid biosynthesis were the most enriched pathways. Among them, biosynthesis of unsaturated fatty acids was the significantly enriched lipid-related metabolic process, and it predominantly appeared in plants rich in proteins, oils, and carbohydrates (59). Moreover, flavonoid biosynthesis and isoflavonoid biosynthesis could be inducible by several abiotic and biotic stimuli (60). Studies have revealed that flavonoids and essential oils are the most abundant constituents in PFL, and we speculated that these three pathways were the main mechanisms of the action of probiotic fermentation on PFL (12, 37). These results further illustrated that in addition to strains and fermentation conditions, the fermentation substrate composition played an important role in metabolic pathway; this might be considered for the biosynthesis of some bioactive metabolites by using probiotics.

## Conclusion

In conclusion, this study employed six different probiotics to ferment PFL, and the dynamic change of the active components and functional activities of PFL during the fermentation process were evaluated. The results showed that short-term fermentation (1–3 days) could significantly improve its chemical components and functional activities. There were significant differences in fermentation performance for different strains, where ATCC 53013 was the best probiotic tool for fermentation PFL. HPLC analysis indicated that rutin, rosmarinic acid, and luteolin were the major compounds with large variations during fermentation. Metabolomics analysis further revealed the differential metabolites including flavonoids, phenols, and fatty acids. KEGG pathway analysis showed that biosynthesis of unsaturated fatty acids, flavonoid biosynthesis, and isoflavonoid biosynthesis were the most enriched metabolic signaling pathways. The results could provide a novel insight into the biotransformation of the active components in natural products, and represent a scientific basis for the further utilization of *Perilla frutescens*. More in-depth studies such as screening more probiotics and optimization of the fermentation process are needed in the following work.

## Data Availability Statement

The original contributions presented in this study are included in the article/Supplementary Material, further inquiries can be directed to the corresponding author/s.

## Author Contributions

ZW and XZ: methodology. ZW and XJ: validation, writing—review, and editing. XZ: formal analysis. ZW: data curation. XJ: writing—original draft preparation. XX: supervision. ZT and XH: project administration. XH: funding acquisition. All authors have read and agreed to the published version of the manuscript.

## Conflict of Interest

The authors declare that the research was conducted in the absence of any commercial or financial relationships that could be construed as a potential conflict of interest.

## Publisher’s Note

All claims expressed in this article are solely those of the authors and do not necessarily represent those of their affiliated organizations, or those of the publisher, the editors and the reviewers. Any product that may be evaluated in this article, or claim that may be made by its manufacturer, is not guaranteed or endorsed by the publisher.

## Footnotes

1
www.kegg.jp/kegg/pathway.html
