# Corrigendum: From function to metabolome: metabolomic analysis reveals the effect of probiotic fermentation on the chemical compositions and biological activities of *Perilla frutescens* leaves

**DOI:** 10.3389/fnut.2024.1357379

**Published:** 2024-01-11

**Authors:** Zhenxing Wang, Ximeng Jin, Xuechun Zhang, Xing Xie, Zongcai Tu, Xiahong He

**Affiliations:** ^1^Key Laboratory for Forest Resources Conservation and Utilization in the Southwest Mountains of China, Ministry of Education, Southwest Forestry University, Kunming, China; ^2^College of Life Sciences, Southwest Forestry University, Kunming, China; ^3^National R&D Center for Freshwater Fish Processing, College of Health, Jiangxi Normal University, Nanchang, China; ^4^College of Horticulture and Landscape, Southwest Forestry University, Kunming, China

**Keywords:** *Perilla frutescens* leaves, fermentation, phytochemical composition, biological activity, metabolomics

In the published article, there was an error. The probiotic name *Lactobacillus Plantarum* SWFU D16 was incorrectly inserted instead of the correct name, *Lacticaseibacillus Paracasei* SWFU D16.

A correction has been made to the **Abstract**. This sentence previously stated:

“PFL was fermented for 7 days using six probiotics (*Lactobacillus Plantarum* SWFU D16, *Lactobacillus Plantarum* ATCC 8014, *Lactobacillus Rhamnosus* ATCC 53013, *Streptococcus Thermophilus* CICC 6038, *Lactobacillus Casei* ATCC 334, and *Lactobacillus Bulgaricus* CICC 6045).”

The corrected sentence appears below:

“PFL was fermented for 7 days using six probiotics (*Lacticaseibacillus Paracasei* SWFU D16, *Lactobacillus Plantarum* ATCC 8014, *Lactobacillus Rhamnosus* ATCC 53013, *Streptococcus Thermophilus* CICC 6038, *Lactobacillus Casei* ATCC 334, and *Lactobacillus Bulgaricus* CICC 6045).”

In the published article, there was an error. The probiotic name *Lactobacillus Plantarum* SWFU D16 was incorrectly inserted instead of the correct name, *Lacticaseibacillus Paracasei* SWFU D16.

A correction has been made to **Materials and methods**, *Chemicals and reagents*, first paragraph. This sentence previously stated:

“*Lactobacillus Plantarum* SWFU D16 was isolated from Yunnan goat milk cake, a Chinese traditional fermented food in our Laboratory.”

The corrected sentence appears below:

“*Lacticaseibacillus Paracasei* SWFU D16 was isolated from Yunnan goat milk cake, a Chinese traditional fermented food in our Laboratory.”

The authors apologize for this error and state that this does not change the scientific conclusions of the article in any way. The original article has been updated.

