# Supplementary figures and images for: From Function to Metabolome: Metabolomic Analysis Reveals the Effect of Probiotic Fermentation on the Chemical Compositions and Biological Activities of Perilla frutescens Leaves
Source: Front Nutr. 2022 Jul 11;9:933193. doi: 10.3389/fnut.2022.933193 (PMC9309800; doi:10.3389/fnut.2022.933193)

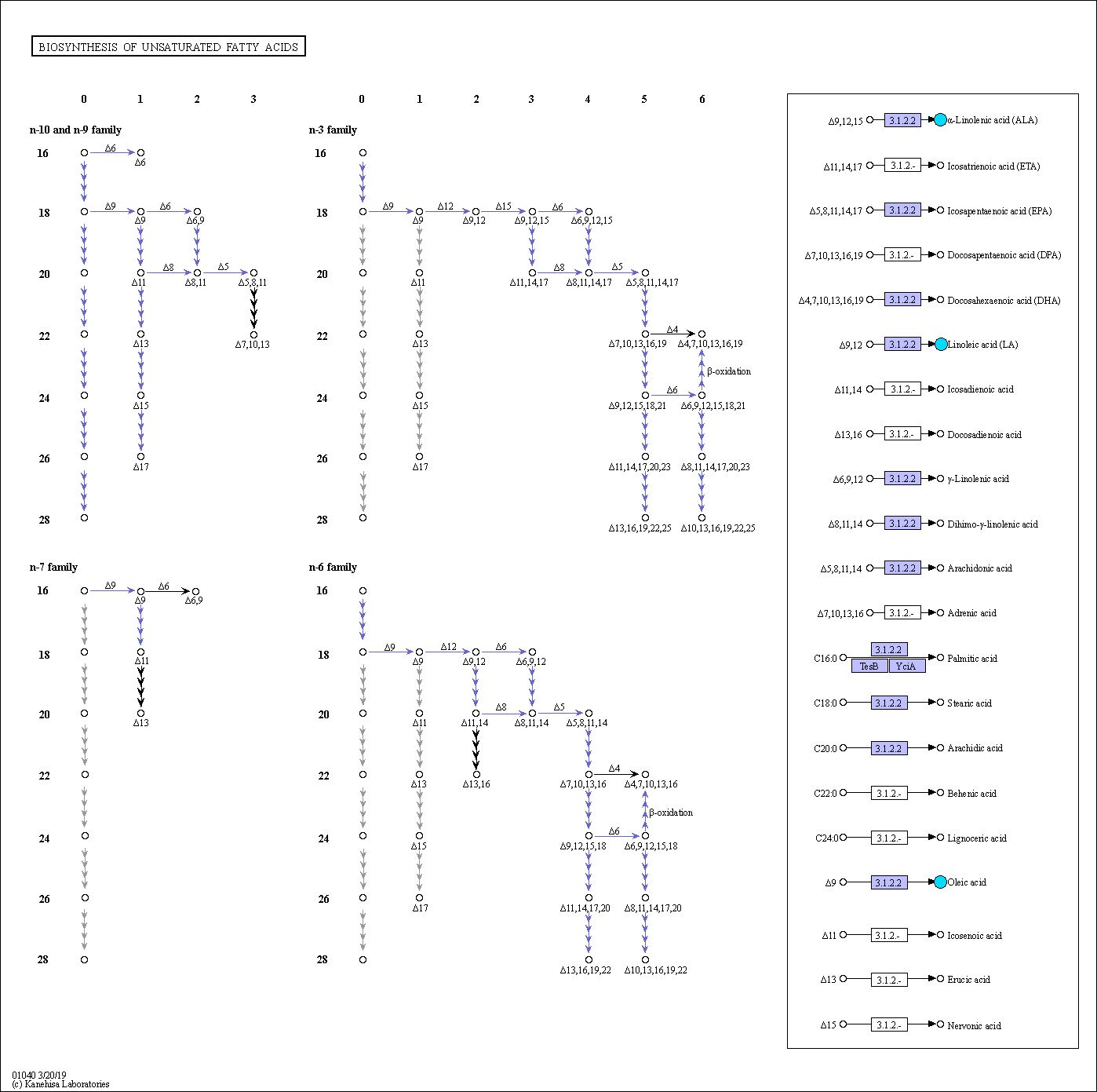

Supplement: Supplementary file 3 [file Image_1.PNG]

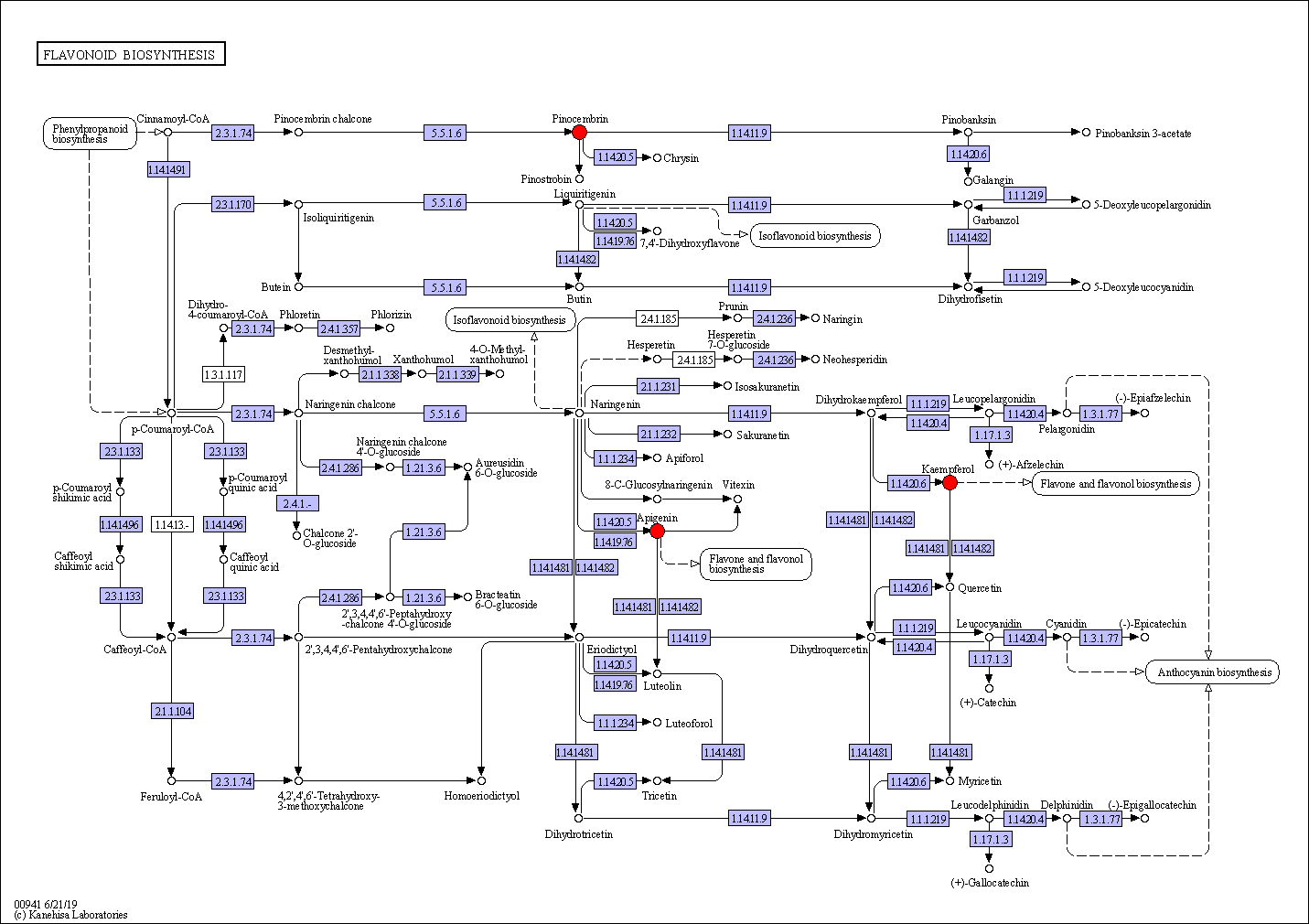

Supplement: Supplementary file 4 [file Image_2.PNG]

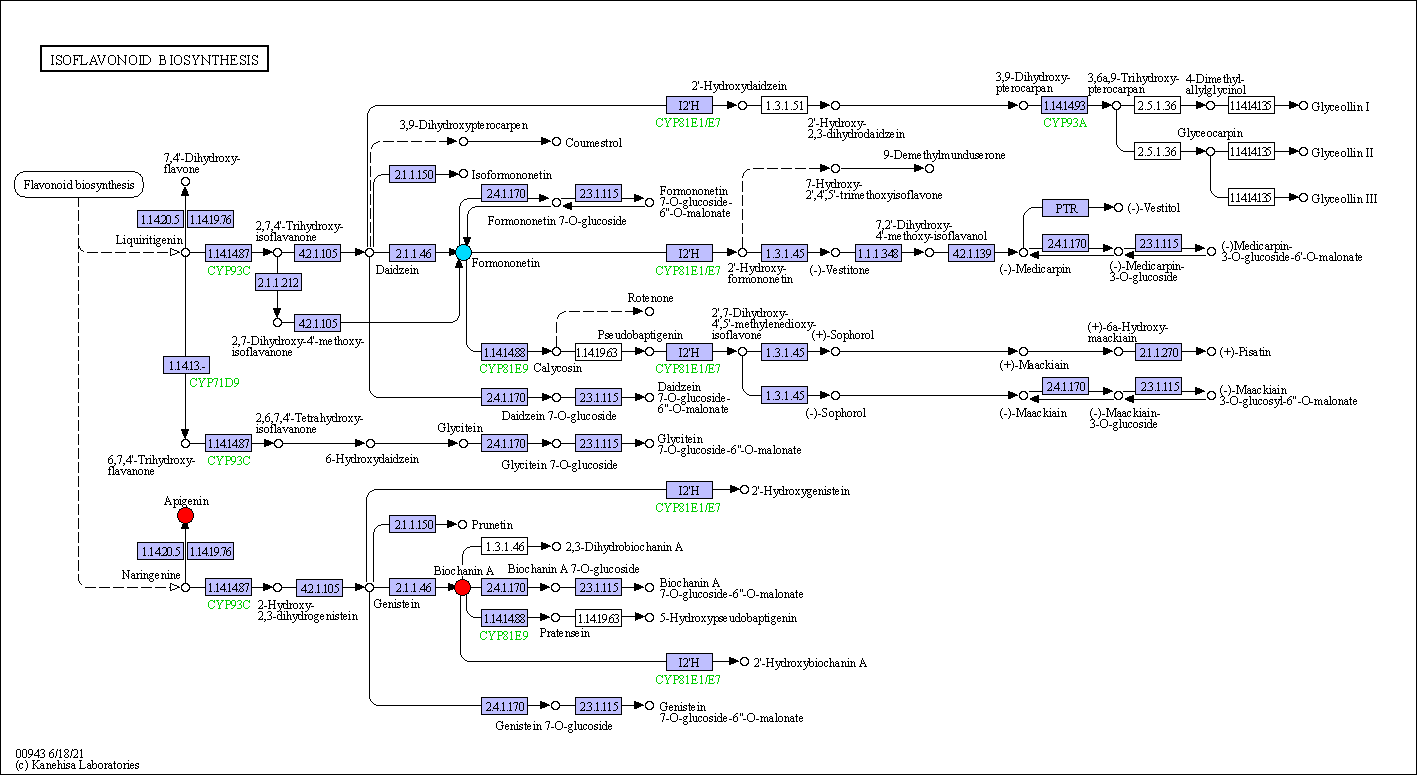

Supplement: Supplementary file 5 [file Image_3.PNG]
